# Chiral Nanoparticle Chains on Inorganic Nanotube Templates

**DOI:** 10.1021/acs.nanolett.3c01213

**Published:** 2023-06-30

**Authors:** Lukáš Kachtík, Daniel Citterberg, Kristýna Bukvišová, Lukáš Kejík, Filip Ligmajer, Martin Kovařík, Tomáš Musálek, Manjunath Krishnappa, Tomáš Šikola, Miroslav Kolíbal

**Affiliations:** † CEITEC BUT, Brno University of Technology, Purkyňova 123, 612 00 Brno, Czech Republic; ‡ Institute of Physical Engineering, Brno University of Technology, Technická 2, 616 69 Brno, Czech Republic; § Faculty of Sciences, Holon Institute of Technology, 52 Golomb St., Holon 5810201, Israel

**Keywords:** nanotubes, nanoparticle assembly, chirality, tungsten disulfide, step edge

## Abstract

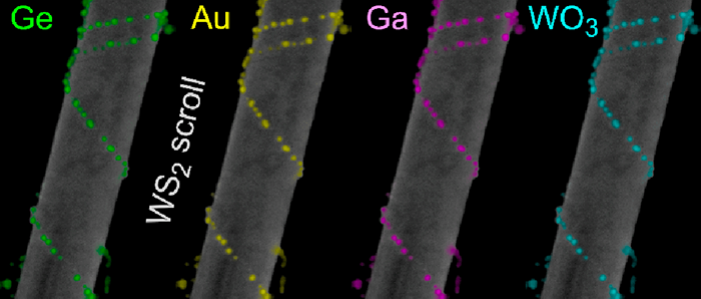

Fabrication of chiral assemblies of plasmonic nanoparticles is a highly attractive and challenging task, with promising applications in light emission, detection, and sensing. So far, primarily organic chiral templates have been used for chirality inscription. Despite recent progress in using chiral ionic liquids in synthesis, the use of organic templates significantly limits the variety of nanoparticle preparation techniques. Here, we demonstrate the utilization of seemingly achiral inorganic nanotubes as templates for the chiral assembly of nanoparticles. We show that both metallic and dielectric nanoparticles can be attached to scroll-like chiral edges propagating on the surfaces of WS_2_ nanotubes. Such assembly can be performed at temperatures as high as 550 °C. This large temperature range significantly widens the portfolio of nanoparticle fabrication techniques, allowing us to demonstrate a variety of chiral nanoparticle assemblies, ranging from metals (Au, Ga), semiconductors (Ge), and compound semiconductors (GaAs) to oxides (WO_3_).

Many important biomolecules and pharmaceuticals are chiral because of the inherent chirality of life on our planet.^1^ The interaction of these chiral molecules with light can be used for discriminating between them, controlling them, or even synthesizing them. The large discrepancy between the size of the molecules and the wavelength of light, however, severely hinders these promising applications. It is therefore desirable to prepare chiral nanostructures and metamaterials that exhibit exceptionally strong light–matter interactions, potentially increasing the sensitivity and throughput wherever the chirality of light–matter interactions comes into question.

The strength of chiroptical effects can be characterized by the dissymmetry factor (*g*-factor), defined as the ratio of differential extinction of circularly polarized light to total extinction. Very large *g*-factors can be reached by squeezing the light waves down to the length scale of biomolecules by carefully designed plasmonic^2,3^ or dielectric^4,5^ nanostructures. The synthesis approaches in this context rely on using chiral seeds or chiral ligands, and thus, chirality can be inscribed into nanoparticles even when achiral building blocks are used^6,7^ and at different length scales.^8^ Their chiroptical response can be further enhanced by inscribing a long-range chiral order to extended clusters of nanoparticles.^9−11^ Such rationally designed assemblies can be tailored to exhibit *g*-factors in the visible part of the electromagnetic spectrum, enhanced by higher-order collective interactions.^12,13^ These chiral superstructures are an attractive candidate for plasmon-enhanced chiral sensing where the single-molecule sensing potential of plasmonics^14^ is utilized in the realm of chirality analysis.^15^ Although there is an ongoing debate whether achiral sensing platforms could be superior to the chiral ones,^16^ the sensing capability of chiral superstructures has already been demonstrated in the detection of markers of Parkinson’s disease^17^ or in the detection of attomolar DNA concentrations.^18^ Going beyond sensing, chiral nanoparticle assemblies can be also used for driving chiral photochemistry and facilitating enantioselective chemical synthesis,^19,20^ which could have a disrupting effect on production of pharmaceuticals. Chiral nanoparticle assemblies could also serve as sources or detectors of circularly polarized light,^21,22^ of dynamic chiral nanomachines,^23^ or in chiral phototherapy.^24^

The strategies used to prepare chiral nanoparticle assemblies comprise two approaches: top-down and bottom-up. The former, usually lithography-based, yield complex geometries^25,26^ but are time-demanding, and it is difficult to scale them up. The latter, on the other hand, utilize either some sort of symmetry breaking during a nanoparticle synthesis process^27−30^ or chirality transfer from an already existing chiral template to achiral nanoparticles.^22,31−33^ The vast majority of the chiral templates are “soft” like, e.g., DNA molecules,^12^ amino acids,^34,35^ micelles,^36^ chiral polymers,^37,38^ chiral ligands in perovskites,^39^ peptides,^40^ or proteins.^17^ Unfortunately, these “soft” templates are fragile and can easily be destroyed at elevated temperatures required in the subsequent synthesis and processing steps. In this respect, chiral ionic liquids are becoming increasingly popular in stabilizing the soft templates at elevated temperatures.^41^ Despite these advancements, the use of soft templates is related to other issues. For example, the bonding flexibility within the soft template can result in changes in interparticle distances (e.g., in a liquid environment) and even disorder of the original helical arrangement.^40^

Utilizing a “hard” inorganic template would naturally offer more degrees of freedom for its further processing steps or functionalization with optically active nanoparticles. However, such synthesis has been demonstrated only using silica nanohelices so far, providing a template for gold^20,42^ or perovskite nanocrystal assemblies.^43^ Moreover, these studies demonstrated only a wet chemistry-based approach, which is limited to in-pot chemical reactions and their products, similar to “soft” templates. Going beyond this approach, here we utilize chiral grain boundaries present on the surface of inorganic nanotubes made of a layered van der Waals material (WS_2_) that serve not only as attachment and nucleation sites for nanoparticles prepared by wet chemistry but also during evaporation under vacuum and high-temperature conditions. The resilience of inorganic nanotubes with respect to high temperatures opens the possibility of bottom-up fabrication of chiral assemblies of nanoparticles made of a much broader group of materials, including those not available so far, like germanium, gallium, or tungsten trioxide.

Line defects (grain boundaries or step edges) on the surfaces of solid-state materials exhibit higher reactivity than the defect-free areas^44,45^ and are thus more vulnerable to chemical attacks such as oxidation or reduction. For example, copper oxide begins to form on a graphene-covered copper foil just beneath the grain boundaries of the polycrystalline graphene, serving as a tool to visualize these otherwise hardly visible boundaries^46^ (see Figure 1a). Notably, crystallographic defects can also be visualized in a similar manner on inorganic nanotubes made of WS_2_^47^ (see Figure 1b). As the smooth surface of nanotubes is mostly inert,^48,49^ the line defects (unsaturated sulfur bonds in the case of WS_2_) are then the only chemically active sites.^50,51^ Due to the tubular shape of the surface, a single-line defect could in principle form a chemically reactive three-dimensional spiral (called a chiral line in the following text), which allows for selective attachment of various molecules or nanoparticles into a helical assembly. To verify this hypothesis, we decorated a large batch of WS_2_ nanotubes with gold nanoparticles using an established one-pot chemistry protocol (see the Methods section for details). During the subsequent inspection, we found that some of the nanotubes indeed exhibit such helical nanoparticle chains (Figure 1c).

**Figure 1:**
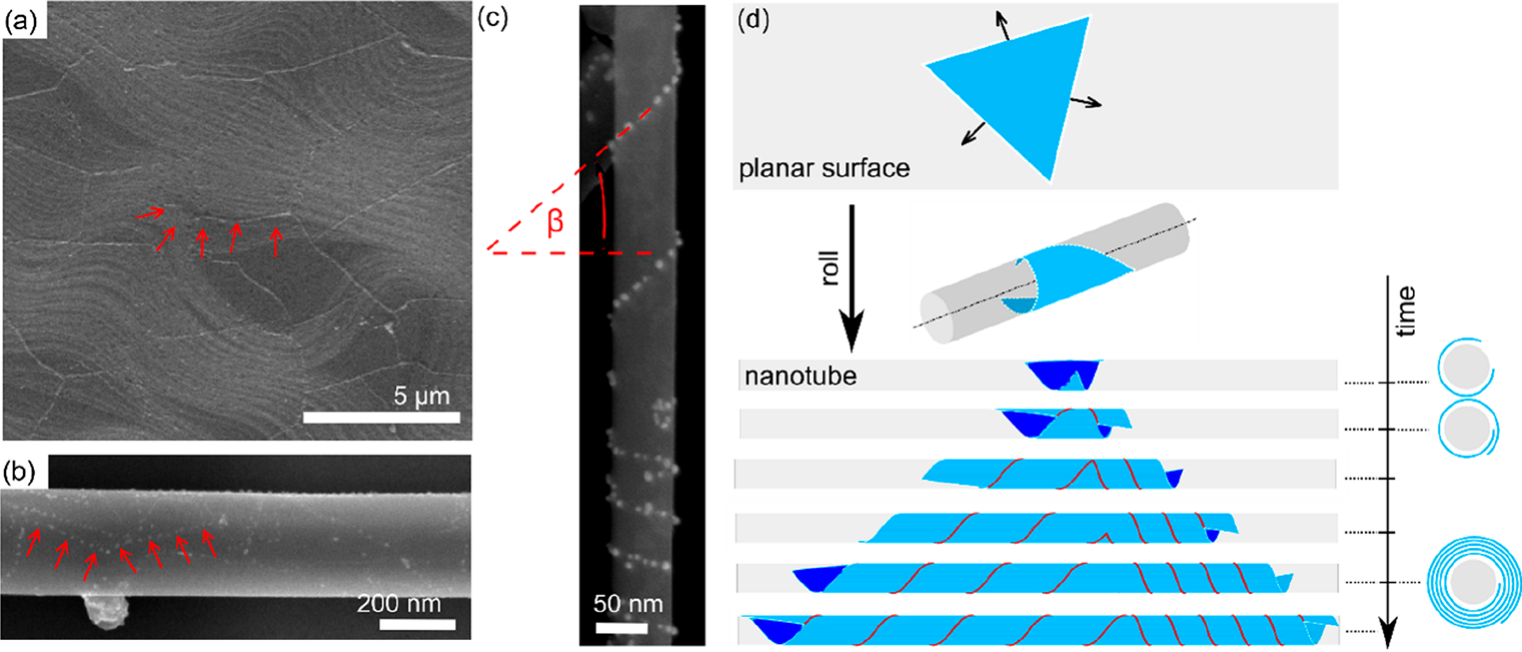
Formation of line defects on tubular templates. (a) Mild oxidation of a flat copper foil covered with polycrystalline graphene reveals graphene’s grain boundaries via formation of copper oxide beneath, as indicated by the red arrows in the scanning electron microscopy (SEM) image. (b) A similar effect is observed on the surface of a WS_2_ nanotube, where the grain boundaries have been accentuated by controlled surface oxidation. (c) SEM micrograph of a WS_2_ nanotube decorated with a helical array of gold nanoparticles. The helix can be characterized by a chiral angle of β, as marked in the image. (d) A theoretical model of lateral growth of a triangular domain on an unwrapped plain surface (top) and on a tubular template (bottom). If the two domain edges meet during growth, one of them overgrows the other, eventually resulting in a scroll-like overlayer. Cross sections at different time intervals are shown as well. See the Supporting Information for more details of the model and also for yet another scenario, where a grain boundary is formed instead of an edge.

In order to understand the nature of the line defect, we have modeled two plausible scenarios of chiral line formation during WS_2_ formation in a growth reactor. Both scenarios describe the same process: sulfurization of tungsten oxide nanowires in H_2_S gas during the formation of WS_2_ nanotubes in a growth reactor.^52−55^ We utilize an analytical model of domain spreading in a tubular geometry (see the Supporting Information for details, Figures S1–S4). For simplicity, we have assumed that growth of an initial WS_2_ domain on the surface of WOx nanowire could occur from only a single nucleation site (see Figure S5). We assume this initial domain to be of triangular shape (Figure 1c) based on the frequent observations in tungsten oxide sulfurization experiments.^56^ The lateral growth of the triangular domain on a cylindrical template inevitably includes collisions of the growing edge with another edge of the very same domain. In the first scenario, the collision leads to formation of a domain boundary, finally resulting in a single helical line defect within an outermost single layer of a nanotube (Figure S5a). This line defect is characterized by a chiral angle, β (see Figure 1c). The final morphology of a decorated nanotube exhibits only a limited range of the chiral angles (see Figures S5b and S6). We will show later that this scenario does not predict all of the experimental results. Nevertheless, it could potentially reproduce line defect formation under different nanotube growth conditions or in different material systems. The second scenario corresponds to overgrowth of an already formed nanotube by an additional WS_2_ layer, e.g., from a supersaturated vapor during the growth reactor cooling down. Figure 1d shows the evolution of growth of such an overlayer. We have again assumed the initial domain to be triangular, which is a common shape resulting from chemical vapor deposition of transition metal dichalcogenides (TMDs) in general and WS_2_ in particular.^57^ In contrast to the first scenario, the propagating layer was allowed to climb over the other edge upon collision and continued to grow laterally as an overlayer (Figure 1d). The resulting step edge then forms the chiral helical line. The final morphology in this scenario is a scroll-like multiwall nanotube (Figure 1d, bottom) instead of a nanotube with in-plane grain boundaries within the outer layer. Notably, there are often two chiral lines corresponding to the two sides of the initial triangular domain. The chiral angles of the two lines are naturally affected by the orientation and position of the initial domain with respect to the nanotube’s primary axis and its ends. The orientation is not restricted by any geometrical or physical constraints, and hence neither are the chiral angles. Furthermore, the geometry of the initial triangular domain dictates that the angle between the two chiral lines is always 60°. These anticipated characteristics match well with the results of nanotube decoration experiments (see Figure S7). Additionally, a transmission electron microscopy (TEM) analysis of the nanotubes decorated with nanoparticle helices confirmed the scroll-like nature of their outer walls (see an example in Figure 2).

**Figure 2:**
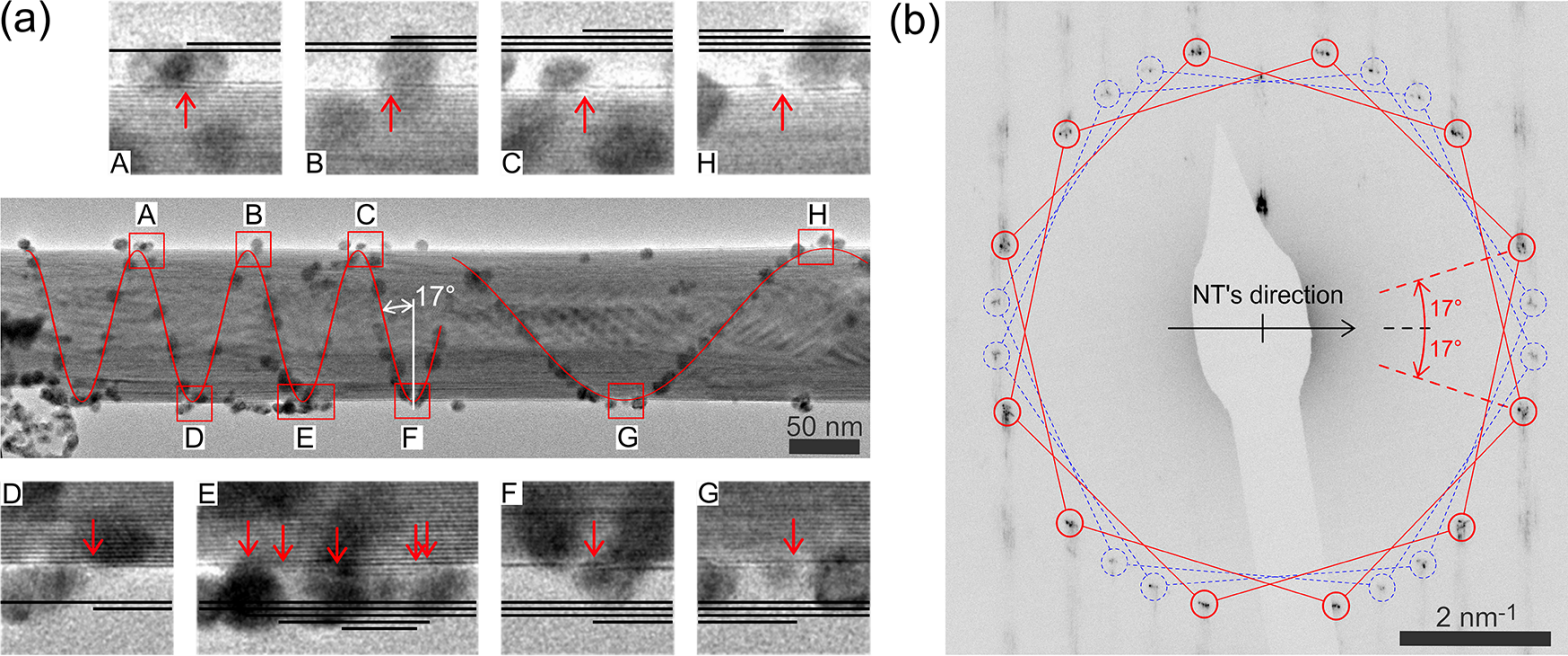
TEM analysis of a WS_2_ nanotube decorated with gold nanoparticles. (a) The TEM image in the center shows a multiwalled WS_2_ nanotube decorated with gold nanoparticles along a chiral line highlighted by the two red sine-shaped curves. Each turning point of the chiral line at the nanotube edge is shown in detail (A–H close-ups). In the close-ups, the individual WS_2_ walls are visible as dark horizontal lines and are highlighted using schematic black lines in sketches above or below the original images. The close-ups confirm that the chiral line is, in fact, a step edge (marked by red arrows) of an outer WS_2_ wall. The close-ups are 25 nm in height. (b) TEM diffraction pattern taken within the A–F region of the same nanotube shown in (a). The pattern comprises two chiral angles (blue and red). One of them (17°) is identical with the one measured from the real space image of the nanoparticle chain (chiral line). The other one (blue) corresponds to the inner wall(s) of the multiwall nanotube.

Figure 2a shows a TEM image of a WS_2_ nanotube decorated with a spiral chain of Au nanoparticles. A detailed inspection of the image reveals noticeable steps at the nanotube’s surface, located precisely at the positions of the chain’s turning points (Figure 2a, close-ups). The step sequence, taken from left to right, consistently exhibits a step-up until a point marked F is reached. From then on, only step-downs are present. Such a sequence indicates that the outer envelope of the nanotube is formed by a rolled wall, which creates a scroll-like tubular surface with a helical chiral line. Every nanotube with a chiral nanoparticle chain we inspected in TEM exposed such a scroll-like overlayer. Such an observation is consistent with the second scenario discussed above (Figure 1d). Figure 2b shows the TEM diffraction pattern of the same nanotube. The diffraction spots can be grouped into two pairs of colored hexagons, where each pair corresponds to a single chiral angle (because the electron beam passes both the top and the bottom part of the nanotube). The existence of multiple hexagons is due to the multiwall nature of the nanotubes, where each inner wall is randomly oriented against each other and can be assigned a chiral angle. However, one of them (17°, red color in Figure 2b) is always identical with that measured in the real space image (Figure 2a). We have similarly analyzed several other nanotubes (see Table S1) and were always able to find a match between the chiral angles measured from the respective diffraction pattern and those measured directly from an SEM image of the nanoparticle chains, thus generalizing our conclusions. A thorough TEM analysis allows us to go even beyond just chiral angle determination: it is possible to identify the nature of step edges (see Figure S8 and Table S1). We have identified them to be of zigzag type, which is consistent with predictive models using density functional theory.^56^ Having identified the chiral lines as step edges instead of grain boundaries makes it possible to utilize the step edge as a nucleation site for adatoms diffusing along the nanotube surface, e.g., during vapor deposition. We demonstrate this capability in the last part of the paper.

Gold nanoparticle assemblies similar to those studied above have been previously demonstrated (albeit with different geometry and dimensions).^12,42^ However, the advantage of our inorganic tubular templates over “soft” organic templates is the possibility of utilizing higher process temperatures; therefore, a wider variety of materials for nanoparticle assemblies can be used. This is demonstrated in Figure 3b, where a WS_2_ nanotube has been controllably oxidized in water vapor at 300 °C,^47^ leading to the formation of WO_3_ nanoparticles along the edge of the outer wall. The very high temperature stability of WS_2_ nanotubes (up to at least 550 °C in high vacuum, as confirmed by a correlative SEM and XPS study)^47^ also offers flexibility in terms of deposition techniques. Figure 3c,d shows WS_2_ nanotubes decorated with gallium and germanium nanoparticles, which were deposited by evaporation of the respective elemental materials under high-vacuum conditions onto nanotubes dispersed over a solid substrate (silicon). Note that gallium could be subsequently oxidized to form gallium oxide, nitrided to form gallium nitride,^58^ or exposed to group V atomic flux to form III–V semiconductor (see Figure S9e, showing XPS analysis of WS_2_ nanotubes decorated by GaAs nanoparticles), thus widening the portfolio of available nanoparticles. There are no limitations other than the deposition temperature that would prevent other techniques like atomic layer deposition^59^ or chemical vapor deposition^60^ from being used for creation of chiral nanoparticle assemblies utilizing these inorganic templates.

**Figure 3:**
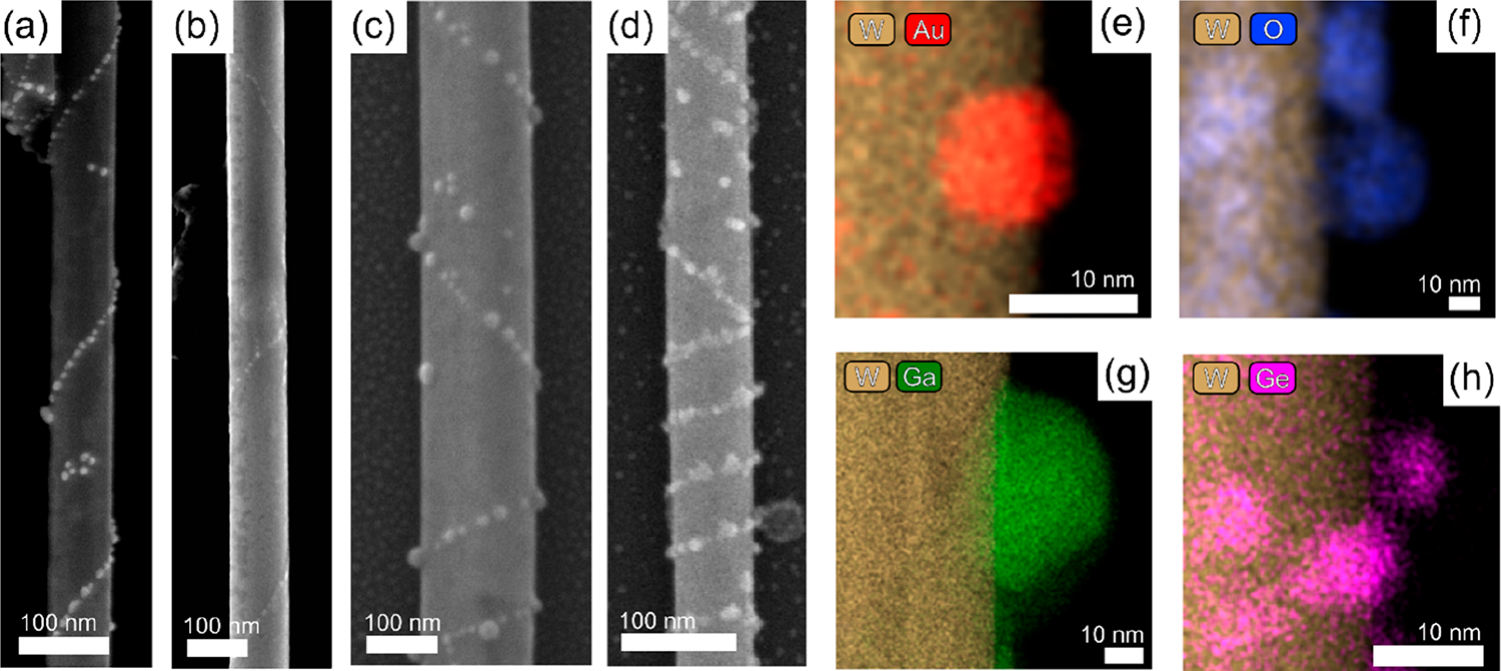
Decoration of chiral WS_2_ nanotubes by various techniques and materials (see the Methods section for details). SEM and elementally filtered EDX images of (a, e) gold-decorated nanotube, resulting from reduction of elemental gold out of an aqueous solution of HAuCl_4_. (b, f) Nanotube decorated with WO_3_, resulting from oxidation by water vapor at 300 °C. (c, g) Gallium- and (d, h) germanium-decorated nanotubes resulting from thermal evaporation of these elemental materials in a vacuum onto dry WS_2_ nanotubes predeposited by drop-casting onto a solid substrate (silicon). For detailed chemical state analysis of the nanoparticles, see Figure S9.

Note that the scroll-like WS_2_ nanotubes discussed here are rarely found within bare unsorted nanotube batches because the synthesis processes for multiwall WS_2_ nanotubes with smooth outer surface are currently well developed.^54^ Here, we have shown that the initially undesired scroll-like products of nanotube synthesis can become templates for further processing toward very attractive nanostructures. Hence, further effort is necessary to identify the growth conditions resulting in the formation of scroll-like nanotubes.. We hypothesize that the scroll-like overlayer forms when a reactor slowly cools from growth temperature during the preparation of WS_2_ nanotube templates. At lower temperatures, it is plausible that the vaporized transient tungsten oxides resulting from the nanotube formation process condense on the outer walls of a few nanotubes and react with the H_2_S residues in the reactor. This CVD-like reaction results in WS_2_ nucleation and overlay growth on the surface of existing WS_2_ nanotubes following the scenario depicted in Figure 1d. Modification of the reactor conditions during cooling could be a possible way toward the formation of scroll-like nanotubes with high yield. Furthermore, precise control over the reactor conditions should allow to restrict the nucleation of the overlayer to the nanotube’s ends and, thus, result in formation of a single helical step edge. This effect is observed even in the current process, although with much less occurrence (see Figure S10). What seems to be difficult to control is the particular handedness. The nanostructures discussed here are inherently racemic; to extract only single handedness, a specific postfabrication sorting technique has to be used. Nevertheless, tubular inorganic templates must not be limited only to nanotubes: nanoscrolls formed by rolling up 2D materials have been experimentally observed since 2003.^61−63^ For example, carbon nanoscrolls can withstand even higher temperatures than TMDs and thus could serve as templates for formation of nanoparticle chains that require even higher synthesis temperatures than shown in this study. Still, despite recent attempts to clarify the rolling process of planar 2D layers, the overall understanding of the formation of the nanoscrolls is still poor.^64,65^ With further advancements in the preparation of these nanostructures, the utilization of the (chiral) defect lines for nanoparticle attachment represents a promising way toward (helical) nanoparticle assemblies of various materials and geometries.

In summary, we have demonstrated the fabrication of metallic, semiconductor, and oxide nanoparticle chiral assemblies by utilizing scroll-like inorganic nanotube templates. In contrast to the commonly used organic templates, WS_2_ nanotubes are stable up to 550 °C in a vacuum. Taking advantage of the very high temperature stability of these templates, we have demonstrated the possibility of preparing a wide range of nanoparticle assemblies (metals, oxides, and semiconductors). Using physical vapor deposition allowed going beyond in-pot chemical synthesis which has been exclusively utilized so far in connection with organic chiral templates.^50,51,66^ Additionally, scroll-like nanotube templates permit to inscribe chirality to otherwise challenging material systems,^67^ like the tungsten trioxide demonstrated here. The versatility of our approach holds promise for the fabrication of chiroptically active building blocks in emerging areas of chiral nanophotonics, where facile fabrication of such assemblies represents a great challenge. The inherent crosstalk between circular and linear dichroisms^68,69^ (which are naturally both present in our decorated nanotubes) prevented us to confirm the characteristic chiral signatures present in optical spectra. With more advance techniques,^70^ such analysis should be possible and could, for example, improve understanding of semiconductor chiroptical nanomaterials that remains elusive, despite their attractiveness in many fields.^71^ Besides, TMD templates covered with plasmonic nanoparticles could become an ideal playground for studying chiral plasmon–exciton polariton complexes.^72^ The proposed fabrication approach thus significantly advances the so-far narrow range of preparation techniques of chiral nanomaterials, allowing for experimental studies of complex chiral systems that have been unavailable up to now.

## Methods

### Synthesis of WS_2_ Inorganic Nanotubes

The WS_2_ nanotubes were prepared via “vapor–gas–solid (VGS)” method, as discussed in detail in our previous reports.^54,55^ In brief, the WO_3_ precursor was initially reduced to tungsten suboxide (WO_2.75_) phase using H_2_ gas as a reducing agent, which results in the formation of 1D WO_2.72_ suboxide nanowhiskers. The as-formed WO_2.72_ nanowhiskers undergo further sulfurization as a “solid–gas” reaction under the continuous flows of H_2_S and H_2_ gases to convert suboxides into multiwalled, hollow tungsten disulfide nanotubes.

### WS_2_ NT-AuNP Complex In-Pot Preparation

We decorated WS_2_ nanotubes with gold nanoparticles using a chemical procedure described in ref (50). Briefly, 2.6 mg of powdered WS_2_ nanotubes was dispersed in 2 mL of isopropyl alcohol (IPA) using a combination of an ultrasonic bath and subsequent mechanical shaking; each step was repeated at least four times. The WS_2_ nanotube dispersion was then added to a hot-plate-heated beaker with a boiling 0.043 mM aqueous solution of HAuCl_4_ under vigorous stirring. The dispersion was still being heated to boil for an additional 3 min, and subsequently the hot plate was turned off and allowed to cool slowly to room temperature. This procedure resulted in WS_2_ nanotubes decorated with gold nanoparticles with an average diameter around 10 nm. By varying the respective ratio between WS_2_ and HAuCl_4_, e.g., by changing the HAuCl_4_ concentration, the average diameter of the AuNP could be tuned between 5 and 30 nm. The resulting dispersion was then drop-casted onto a cleaned substrate (silicon wafer, glass covered with an indium tin oxide film, or TiN membrane).

### WS2 NT: Other NP Complexes by Evaporation under Vacuum Conditions

As described above, the nanotubes were dispersed in IPA using an ultrasonic bath and mechanical shaking. Next, the solution was drop-casted onto a silicon substrate and allowed to dry. The resulting sample was inserted into a complex of ultrahigh-vacuum chambers (base pressure *p* = 1 × 10^–10^ mbar) equipped with gallium, germanium, and arsenic evaporators. The sample was first annealed using a calibrated pBN heating element at 350 °C for 20 min to degas any IPA residues and then stabilized at growth temperature (215 and 360 °C for gallium and germanium, respectively). The growth temperature, flux, and overall duration were optimized to achieve nanoparticle formation on the chiral line only. For gallium, the optimum flux was 1.08 nm/min for 2 min. For germanium, the optimum flux was 0.05 nm/min for 10 min. In the case of droplet epitaxy demonstrated in Figure S9e, the WS_2_ nanotubes with Ga nanoparticles were exposed to As flux (equivalent to 2.5 × 10^–5^ Pa) at elevated temperature (215 °C) for an additional 1 h.

### Chemical Characterization

For EDX analysis, the NTs (either with in-pot-prepared Au NPs or just bare) were dispersed onto a standard 300-mesh Mo grid. In the latter case, the dispersion was followed by evaporation of specific chemical element under vacuum or by oxidation to get WO_3_ NPs. The resulting structures were characterized by a Thermo Fisher Talos TEM, equipped with Dual-X and Super-X EDX detectors. For XPS, the NTs were dispersed on a silicon substrate, again followed by a respective procedure. The subsequent analysis was performed either without breaking vacuum conditions by a SPECS Phoibos 150 system (Ga) or ex situ by a Kratos Axis Supra (WO_3_, Au, Ge). The peak fitting details as well as XPS acquisition conditions are described in the Supporting Information.

## Supporting Information Available

Mathematical model of 2D layer formation on the cylindrical surface; additional SEM images; explanation of TEM chirality analysis and step-edge termination; handedness statistics; XPS spectra and acquisition conditions (PDF)
