# Supplementary material for: Chiral Nanoparticle Chains on Inorganic Nanotube Templates
Source: Nano Lett. 2023 Jun 30;23(13):6010–7. doi: 10.1021/acs.nanolett.3c01213 (PMC10347696; doi:10.1021/acs.nanolett.3c01213)
Supplement: Supplementary file 1 — nl3c01213_si_001.pdf [file nl3c01213_si_001.pdf]

Supporting Information

for

Chiral nanoparticle chains on inorganic nanotube templates

Lukáš Kachtík[1,ǁ], Daniel Citterberg[1,ǁ], Kristýna Bukvišová[1], Lukáš Kejík[1], Filip Ligmajer[1,2], Martin Kovařík[1,2], Tomáš Musálek[1,2], Manjunath Krishnappa[3], Tomáš Šikola[1,2] and Miroslav Kolíbal[1,2]*

[1]CEITEC BUT, Brno University of Technology, Purkyňova 123, 612 00 Brno, Czech Republic

[2]Institute of Physical Engineering, Brno University of Technology, Technická 2, 616 69 Brno, Czech Republic

[3]Faculty of Sciences, Holon Institute of Technology, 52 Golomb St., Holon, 5810201, Israel

ǁ contributed equally to this work

*kolibal.m@fme.vutbr.cz

Table of contents

1. Mathematical model of 2D layer formation on the cylindrical surface……………………….2

Fig. S5: Formation of a grain boundary line defect on a tubular template……..………...…..10

Fig. S6: Dependency of the chiral angle on the rotation of the domain …………………….…11

Fig. S7: Outer layer morphology visualized by gold nanoparticle attachment ……………..…..12

Fig. S8: Explanation of TEM analysis of nanotubes' chirality ….…………………………..13

Table S1: Determination of chirality of the outer layer and step-edge termination ……….....14

Fig. S9: Explanation of TEM analysis of nanotubes' chirality ….…………………………..15

Fig. S10: Chemical state of the nanoparticles shown in Fig. 3 and XPS analysis of GaAs nanoparticles prepared by droplet epitaxy ……..……………..…..…………………………..17

1. Mathematical model of 2D layer formation on the cylindrical surface

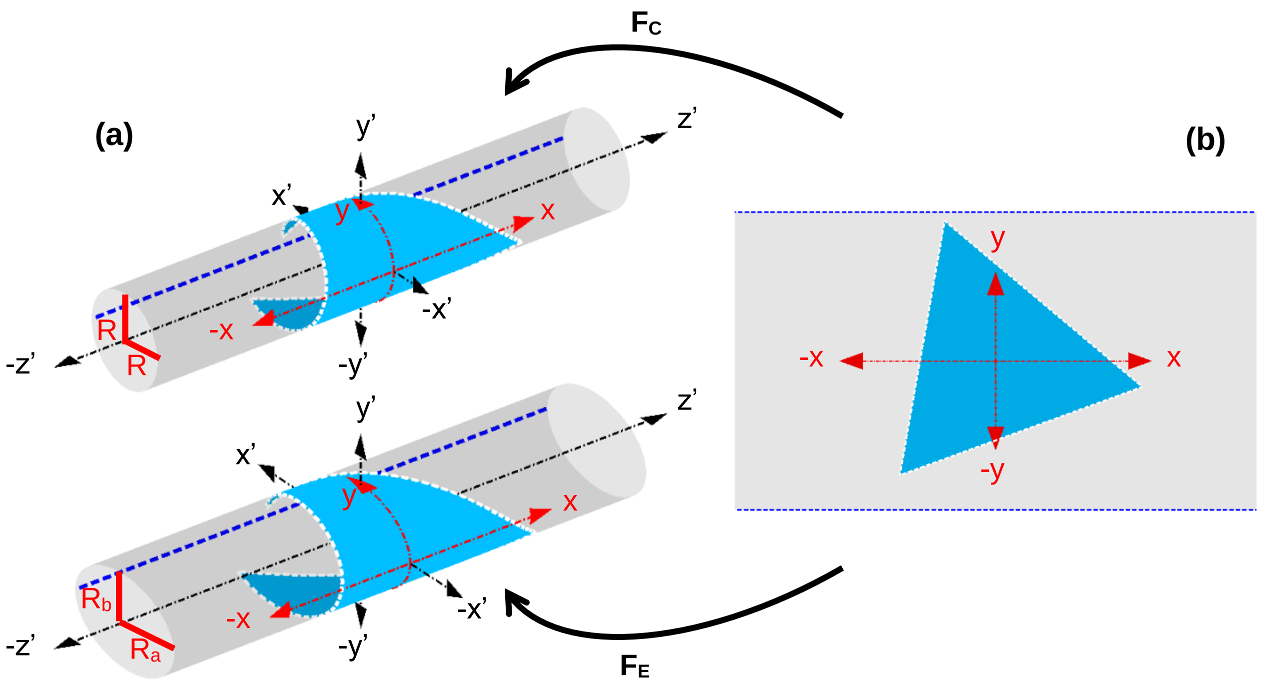

Fig. S1: Triangular WS_2 domain on top of the surface of the cylindrical NT (a) and domain mapped in 2D plane (b). (a) shows a WS_2 domain in form of an equilateral triangle on top of the NT with circular cross-section and radius R (up) and domain on top of the NT with elliptical cross-section with axis lengths R_a and R_b. (down). F_C and F_E symbolize the transformation equations from 2D plane onto surface of the NT (equation 1.1). **(b)** shows an equilateral triangle (growing WS_2 domain) in 2D plane.

Growth of a single WS_2 domain on top of the NT is firstly simplified by the assumption of the preexisting nanotube in form of the cylinder, on top of which a WS_2 domain is spreading uninterruptedly until it collides with itself. Based upon the observation of the WS_2 monolayers growth (Ref. 52 in the main article), a shape of the domain is chosen to be an equilateral triangle.

First step is to reduce a complexity of the proposed problem from 3D to 2D. Mapping of the nanotube's surface depends on the shape of cylinder's base. After the mapping of the surface, a solution will be simplified to equilateral triangle spreading in the 2D plane. Mappings (see **Fig. S1**) of the 2D plane (with coordinates $[x, y]$) onto the cylinders' surfaces (with coordinates $[x', y', z']$) with circular base F_C and elliptical base F_E are following:

$$\begin{matrix} x \\ y \end{matrix} \xrightarrow{F_C} \begin{matrix} x' = -R\cos\dfrac{y}{R} \\ y' = R\sin\dfrac{y}{R} \\ z' = x \end{matrix} \quad \Bigg| \quad \begin{matrix} x \\ y \end{matrix} \xrightarrow{F_E} \begin{matrix} x' = -R_a\cos\dfrac{y}{R_a} \\ y' = R_b\sin\dfrac{y}{R_b} \\ z' = x \end{matrix} \qquad (1.1).$$

The trigonometric functions contained in the mapping equations ensure, that any portion of the domain exceeding blue dashed line in 2D plane (**Fig. S1(b)**) will still be mapped onto the surface of the nanotube in 3D. Henceforth, it is enough to investigate the growing domain in 2D plane with additional condition characterizing possible self-collision of the domain. An unconstrained domain growth needs to be introduced first. Shape of the domain is

chosen to be triangular. A triangle with sides expanding in the 2D plane can be defined in the following way:

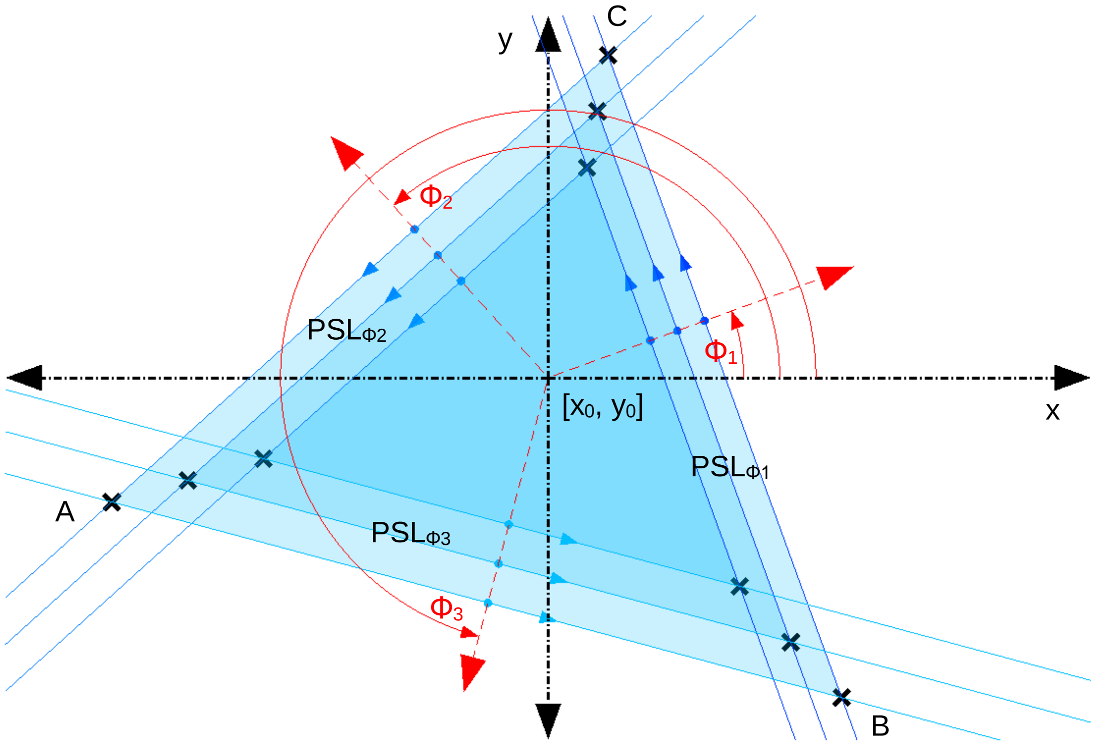

Fig. S2: **Construction of the triangular WS_2 domain.** WS_2 domain is constructed from three lines PSL_{ϕ_i} each perpendicular to the direction of the spreading ϕ_i. To construct such lines (domain sides) the points LP_{ϕ_i} moving in direction ϕ_i with increasing time from nucleation center $[x_0, y_0]$ are found (dots in different shades of blue). After finding unit vectors of these lines LV_{ϕ_i} (arrows in different shades of blue) a domain sides are defined. Domain vertices (black crosses) are found as the crossing points of two of the lines PSL_{ϕ_i}. For future computations, the vertices of the triangle are labeled as A, B and C. The image was generated with parameters $\phi_1 = 20°$, $\phi_2 = 132°$, $\phi_3 = 255°$, $v_1 = 1$, $v_1 = 1.2$ and $v_1 = 1.4$ (speed is in normalized units: unit distance per unit time) in three time steps: $t_1 = 1.5$, $t_2 = 1.9$ and $t_3 = 2.3$ (time is in arbitrary units).

Three lines – defining the sides of the triangle – are constructed. Each line is defined by two parameters: a direction angle ϕ_i into which the line is spreading and the speed of the spreading line (triangle side) v_i (**Fig. S2**). Velocities of lines movements are defined as following:

$$\boldsymbol{v}_{\boldsymbol{\phi}_1} = v_1 \cdot [\cos\phi_1, \sin\phi_1]$$
$$\boldsymbol{v}_{\boldsymbol{\phi}_2} = v_2 \cdot [\cos\phi_2, \sin\phi_2] \quad (1.2),$$
$$\boldsymbol{v}_{\boldsymbol{\phi}_3} = v_3 \cdot [\cos\phi_3, \sin\phi_3]$$

where v_i is speed of movement in direction ϕ_i. To define such lines, a slope of the line and point belonging to the line need to be found. Resulting from the fact that line will be perpendicular to the direction of the spreading a unit vector (**Fig. S2**), one can write:

$$\begin{aligned}
\mathrm{LV}_{\phi_1} &= \left[\mathrm{LV}_{x,\phi_1}, \mathrm{LV}_{y,\phi_1}\right] = \left[\cos\left(\phi_1 + \frac{\pi}{2}\right), \sin\left(\phi_1 + \frac{\pi}{2}\right)\right] = [-\sin\phi_1, \cos\phi_1] \\
\mathrm{LV}_{\phi_2} &= \left[\mathrm{LV}_{x,\phi_1}, \mathrm{LV}_{y,\phi_1}\right] = \left[\cos\left(\phi_2 + \frac{\pi}{2}\right), \sin\left(\phi_2 + \frac{\pi}{2}\right)\right] = [-\sin\phi_2, \cos\phi_2] \quad (1.3). \\
\mathrm{LV}_{\phi_3} &= \left[\mathrm{LV}_{x,\phi_1}, \mathrm{LV}_{y,\phi_1}\right] = \left[\cos\left(\phi_3 + \frac{\pi}{2}\right), \sin\left(\phi_3 + \frac{\pi}{2}\right)\right] = [-\sin\phi_3, \cos\phi_3]
\end{aligned}$$

The point is defined as distance from center $[x_0, y_0]$ traveled with speed v_i in direction ϕ_i during time t (in time $t = 0$ the lines are collapsed into single point $[x_0, y_0]$ – nucleation center):

$$\begin{aligned}
\mathrm{LP}_{\phi_1} &= \left[\mathrm{LP}_{x,\phi_1}, \mathrm{LP}_{y,\phi_1}\right] = [x_0 + v_1 t\cos\phi_1, y_0 + v_1 t\sin\phi_1] \\
\mathrm{LP}_{\phi_2} &= \left[\mathrm{LP}_{x,\phi_2}, \mathrm{LP}_{y,\phi_2}\right] = [x_0 + v_2 t\cos\phi_2, y_0 + v_2 t\sin\phi_2] \quad (1.4). \\
\mathrm{LP}_{\phi_3} &= \left[\mathrm{LP}_{x,\phi_3}, \mathrm{LP}_{y,\phi_3}\right] = [x_0 + v_3 t\cos\phi_3, y_0 + v_3 t\sin\phi_3]
\end{aligned}$$

Now, spreading lines are constructed in a Point-Slope form of the equation of a straight line: $(y - y_{\mathrm{KP}}) = k(x - x_{\mathrm{KP}})$, where k is a slope of the line

$$k = \frac{\mathrm{LV}_{y,\phi_i}}{\mathrm{LV}_{x,\phi_i}} = -\frac{\cos\phi_i}{\sin\phi_i} = -\cot\phi_i \quad (1.5),$$

and known point $[x_{\mathrm{KP}}, y_{\mathrm{KP}}]$ is LP_{ϕ_i}. Resulting line equations take following form:

$$\begin{aligned}
\mathrm{PSL}_{\phi_1}&: \quad y = -x\cot\phi_1 + x_0\cot\phi_1 + v_1 t\cot\phi_1\cos\phi_1 + y_0 + v_1 t\sin\phi_1 \\
\mathrm{PSL}_{\phi_2}&: \quad y = -x\cot\phi_2 + x_0\cot\phi_2 + v_2 t\cot\phi_2\cos\phi_2 + y_0 + v_2 t\sin\phi_2 \quad (1.6). \\
\mathrm{PSL}_{\phi_3}&: \quad y = -x\cot\phi_3 + x_0\cot\phi_3 + v_3 t\cot\phi_3\cos\phi_3 + y_0 + v_3 t\sin\phi_3
\end{aligned}$$

As a next step, the vertices of the triangle can be found as a crossing point of two of the lines. If no two lines are parallel, there is always exactly one crossing point in a 2D plane – one of the vertices of the triangle. After simplification the vertices of the triangle are following:

$$\begin{aligned}
\mathrm{A} &= \left[\mathrm{A}_x, \mathrm{A}_y\right] = [x_0 + t a_x, y_0 + t a_y] \\
\mathrm{B} &= \left[\mathrm{B}_x, \mathrm{B}_y\right] = [x_0 + t b_x, y_0 + t b_y] \quad (1.7), \\
\mathrm{C} &= \left[\mathrm{C}_x, \mathrm{C}_y\right] = [x_0 + t c_x, y_0 + t c_y]
\end{aligned}$$

$$\begin{aligned}
a_x &= \frac{v_3\sin\phi_2 - v_2\sin\phi_3}{\sin(\phi_2 - \phi_3)} \quad b_x = \frac{v_1\sin\phi_3 - v_3\sin\phi_1}{\sin(\phi_3 - \phi_1)} \quad c_x = \frac{v_2\sin\phi_1 - v_1\sin\phi_2}{\sin(\phi_1 - \phi_2)} \\
a_y &= \frac{v_2\cos\phi_3 - v_3\cos\phi_2}{\sin(\phi_2 - \phi_3)} \quad b_y = \frac{v_3\cos\phi_1 - v_1\cos\phi_3}{\sin(\phi_3 - \phi_1)} \quad c_y = \frac{v_1\cos\phi_2 - v_2\cos\phi_1}{\sin(\phi_1 - \phi_2)}
\end{aligned} \quad (1.8).$$

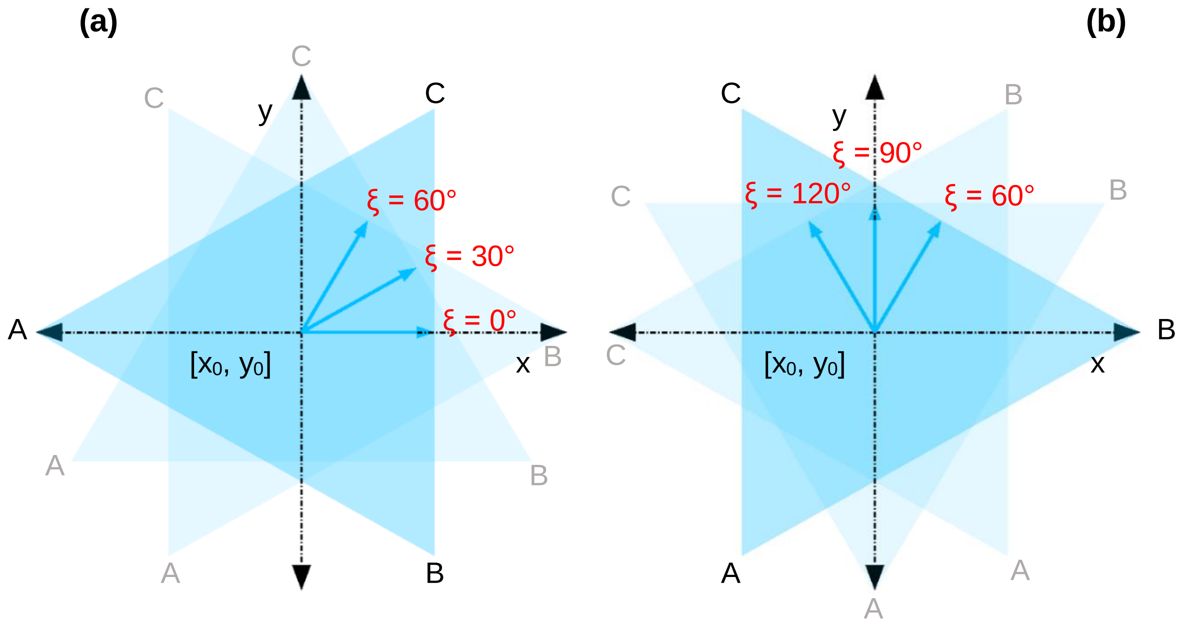

Fig. S3: **Examples of the different rotations of the equilateral-triangle WS_2 domain. (a)** shows examples of the **case 1,** where $\xi_\epsilon(0°, 60°)$. During the domain growth the vertex C will collide with domain side $\overline{AB}$ and subsequently form a defect line upon additional domain growth. **(b)** shows domain rotations of the **case 2,** where $\xi_\epsilon(60°, 120°)$. In this case, during the domain growth the domain vertex A will collide with domain side $\overline{BC}$ forming defect line upon additional domain growth.

From now on a triangle is defined by its vertices. To acquire an equilateral triangle, the direction angles ϕ_i follow equations:

$$\begin{aligned}\phi_1 &= \xi \\ \phi_2 &= \xi + 120° \quad (1.9), \\ \phi_3 &= \xi + 240°\end{aligned}$$

where ξ is the rotation angle of the equilateral triangle with respect to the x axis (**Fig. S3**). Vertices of the triangle are indistinguishable from each other (notation A, B, C serves only for computational purposes) so the rotation angles $\xi = 120°$ and $\xi = 240°$ give the identical triangle to $\xi = 0°$. Thanks to the symmetry of the problem, it is sufficient to investigate only rotation angles from interval: $\xi_\epsilon(0°, 120°)$. A short notice on limiting case of $\xi = 0°$ (or $\xi = 60°$) will be made later in text. In the next paragraph, the conditions of self-collision will be investigated.

It can be seen from **Fig. S1** and equations 1.1 that two points from 2D plane will occupy the same place in 3D (collide with each other) if their x coordinate is the same and y-distance of these points d_y is equal to the value of the perimeter of the cylinder base ($d_P = 2\pi R$ for circular base and $d_P = 4R_a E(k)$ for elliptic base, if $R_a > R_b$ and where $E(k)$ is complete elliptic integral of the second kind with argument $k = \sqrt{1 - (R_b/R_a)^2}$). Here, the solution is split into two cases (**Fig. S4**): **case 1** is for $\xi_\epsilon(0°, 60°)$ and **case 2** is for $\xi_\epsilon(60°, 120°)$. In both cases, two chiral (boundary) lines are created. As the domain (triangle) is growing, one of the vertices will eventually collide with the side of the domain. As the triangular domain will spread further, more collisions of the domain with itself will occur forming a defect line – one to the left of the initial crossing point and one to the right. Equations characterizing these defect lines can be found below. The straight defect line in 2D will form a spiral on top of cylindrical nanotube in 3D and will be called chiral line in further text.

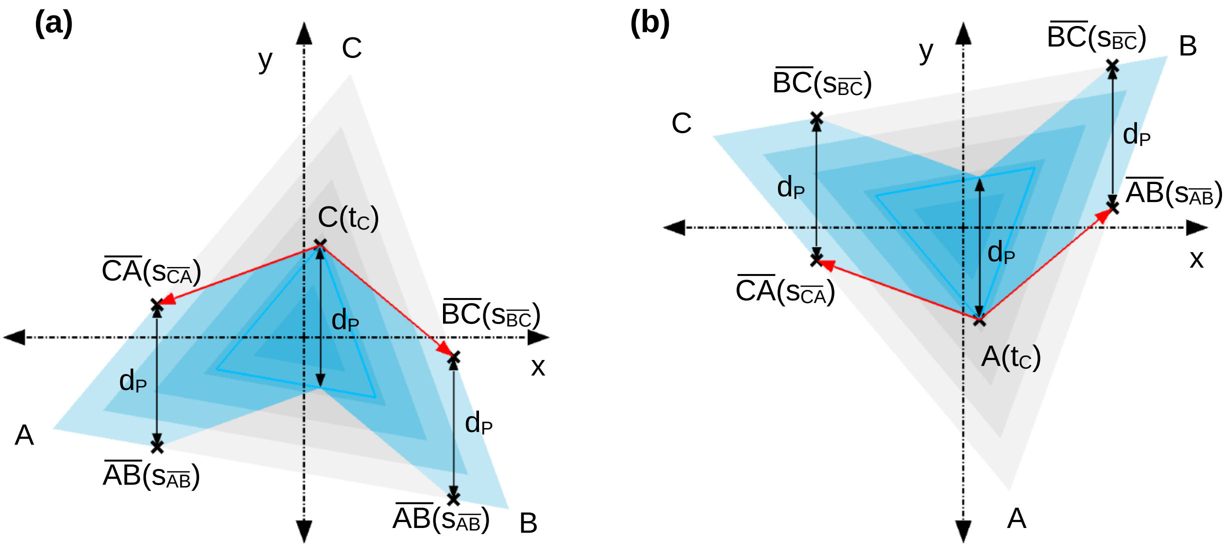

Fig. S4: **Collisions of the WS_2 domain with itself.** Both images show five time-steps of the growing WS_2 domain. Grey equilateral triangle shows a hypothetical case of how the domain would look without self-collisions on the cylinder, and blue polygons show the domain's shape after colliding with itself. A moment of the first collision in time t_c is highlighted. Also, a y-distance equal to d_P in two different time steps is highlighted (one for time t_c and one for time $t > t_c$ for left and right chiral line). Moreover, the other significant points of collisions are highlighted with black crosses (eq.: 1.15, 1.16, 1.19, 1.20, 1.24 and 1.25). **(a)** shows **case 1**, where vertex C collides with domain side $\overline{AB}$. Left chiral line is created by collision of the domain sides $\overline{AB}$ and $\overline{CA}$ and right chiral line arises from the collision of the domain sides $\overline{AB}$ and $\overline{BC}$. **(b)** shows **case 2**, where vertex A collides with domain side $\overline{BC}$. Left chiral line comes from collision of the domain sides $\overline{BC}$ and $\overline{CA}$ and right chiral line is created by the collision of the domain sides $\overline{AB}$ and $\overline{BC}$.

Case 1: In the range of rotation angles $\xi_\epsilon(0°, 60°)$ the vertex C of the domain will collide with the domain side $\overline{AB}$. The exact time of this collision can be found as follows. First, domain side $\overline{AB}$ is written as parametric equation of the straight-line segment:

$$\overline{AB}: \quad \begin{aligned} x &= A_x + s(B_x - A_x) \\ y &= A_y + s(B_y - A_y) \\ s_\epsilon &\langle 0,1 \rangle \end{aligned} \quad (1.10).$$

Point of the domain side $\overline{AB}$ colliding with vertex C can be found by applying condition $C_x = x$ (where C_x is x component of vertex C from equations 1.7/1.8 and x is from equation 1.10). After simplification, an s parameter satisfying the equation is found:

$$s_{\overline{AB}} = \frac{a_x - c_x}{a_x - b_x} \quad (1.11).$$

Now the y-distance d_y of the vertex C and point on the domain side $\overline{AB}$ can be defined:

$$d_y = C_y - y(s_{\overline{AB}}) \quad (1.12),$$

where C_y is y component of the vortex C from equation 1.7. When y-distance of the vertex C and domain side $\overline{AB}$ is equal to the value of the perimeter of the cylinder base, $d_y = d_P$, C

vertex will collide with the domain side $\overline{\mathrm{AB}}$. In the interval of the rotation angles $\xi_\epsilon(0°, 60°)$ it will be always point C colliding with the domain side $\overline{\mathrm{AB}}$. Additionally, y-distance d_y defined as 1.12 will be a positive number. Combining equations 1.10 and 1.11 and substituting the values into 1.12 with $d_y = d_P$, a collision time t_c is acquired:

$$t_c = d_P \frac{\sin\phi_3 \sin(\phi_1 - \phi_2)}{v_1 \sin(\phi_3 - \phi_2) + v_2 \sin(\phi_1 - \phi_3) + v_3 \sin(\phi_2 - \phi_1)} \quad (1.13).$$

After this time, expanding domain will continue to collide with itself forming chiral lines left to the vertex C and right to the vertex C (**Fig. S4(a)**). To find equations of these chiral lines, a similar approach is implemented. For a left defect line, a domain side $\overline{\mathrm{AB}}$ (eq.: 1.10) will be colliding with the side $\overline{\mathrm{CA}}$:

$$\overline{\mathrm{CA}}: \quad \begin{array}{c} x = \mathrm{C}_x + s(\mathrm{A}_x - \mathrm{C}_x) \\ y = \mathrm{C}_y + s(\mathrm{A}_y - \mathrm{C}_y) \\ s_\epsilon\langle 0,1\rangle \end{array} \quad (1.14).$$

First, s parameters are found. Forcing x coordinates from equation 1.10 and 1.14 to be equal, a relation between s parameters is found:

$$s_{\overline{\mathrm{AB}}} = \frac{c_x - a_x + s_{\overline{\mathrm{CA}}}(a_x - c_x)}{(b_x - a_x)} \quad (1.15),$$

with coefficients from eq. 1.8. Further employing y-distance as subtraction of the y coordinates from equations 1.14 and 1.10, a s parameter of the point on domain side $\overline{\mathrm{CA}}$ can be found so the y-distance of this point from point on domain side $\overline{\mathrm{AB}}$ is d_P:

$$s_{\overline{\mathrm{CA}}} = \frac{\left(\frac{d_P}{t} - c_y + a_y\right)(b_x - a_x) + (c_x - a_x)(b_y - a_y)}{(a_y - c_y)(b_x - a_x) - (a_x - c_x)(b_y - a_y)} \quad (1.16).$$

Substituting this value back to the equations 1.14 and simplifying the equations, a parametric representation of the left defect line LDL is acquired:

$$\mathrm{LDL}: \quad \begin{array}{c} x = \mathrm{A}_x - d_P \dfrac{\sin\phi_2 \sin\phi_3}{\sin(\phi_3 - \phi_2)} \\ y = \mathrm{A}_y + d_P \dfrac{\cos\phi_2 \sin\phi_3}{\sin(\phi_3 - \phi_2)} \end{array} \quad (1.17).$$

This is the defect line created from points of the domain side $\overline{\mathrm{CA}}$. In 2D there is a left defect line also constructed from points of the domain side $\overline{\mathrm{AB}}$ but it has the same x coordinate, and d_P value is subtracted from y coordinate. These two lines in 3D occupy the same place, so for the further computations only one of them is relevant.

The same approach is applied to construct right chiral line RDL. The chiral line is constructed as a crossing of the domain sides $\overline{\mathrm{AB}}$ and $\overline{\mathrm{BC}}$, having parametric representation:

$$\overline{\mathrm{BC}}: \quad \begin{array}{c} x = \mathrm{B}_x + s(\mathrm{C}_x - \mathrm{B}_x) \\ y = \mathrm{B}_y + s(\mathrm{C}_y - \mathrm{B}_y) \\ s_\epsilon\langle 0,1\rangle \end{array} \quad (1.18).$$

Implementing the same reasoning as for LDL, the s parameter is:

$$s_{\overline{\mathrm{AB}}} = \frac{b_x - a_x + s_{\overline{\mathrm{BC}}}(c_x - b_x)}{b_x - a_x} \quad (1.19),$$

$$s_{\overline{\mathrm{BC}}} = \frac{\left(\frac{d_P}{t} - b_y + a_y\right)(b_x - a_x) + (b_x - a_x)(b_y - a_y)}{(c_y - b_y)(b_x - a_x) - (c_x - b_x)(b_y - a_y)} \quad (1.20).$$

After substituting eq. 1.20 into eq. 1.18, a parametric representation of the RDL is acquired:

$$\text{RDL:} \quad \begin{aligned} x &= \mathrm{B}_x - d_P \frac{\sin\phi_1 \sin\phi_3}{\sin(\phi_3 - \phi_1)} \\ y &= \mathrm{B}_y + d_P \frac{\cos\phi_1 \sin\phi_3}{\sin(\phi_3 - \phi_1)} \end{aligned} \quad (1.21).$$

Now the directions in which these chiral lines are spread can be found. Looking at the point of the chiral line in time t and subsequent time $t + dt$, it can be written:

LDL: **RDL**:

$$\alpha_{ll} = \operatorname{atan}\frac{y(t+dt) - y(t)}{x(t+dt) - x(t)} = \operatorname{atan}\frac{a_y}{a_x} \quad \alpha_{rl} = \operatorname{atan}\frac{y(t+dt) - y(t)}{x(t+dt) - x(t)} = \operatorname{atan}\frac{b_y}{b_x} \quad (1.22)$$

with x, y coming from the eq. 1.17 for LDL and eq. 1.21 for RDL and coefficients from eq. 1.8. From eq. 1.22 it can be deduced that left chiral line is spreading in the direction of vortex A and right chiral line is spreading in the direction of vortex B. Now the process needs to be repeated for case 2 to cover all possibilities. The only omitted scenarios are those for $\xi = 0°$ and $\xi = 60°$. In these two scenarios, one side of the domain is spreading in the direction of the long axis of the nanotube (in $\pm z'$ direction from **Fig. S1**). Hence, no chiral line is formed, or the chiral line is parallel to the long axis of the nanotube.

Case 2: In the range of rotation angles $\xi_\epsilon(60°, 120°)$ the vortex A of the domain will collide with the domain side $\overline{\mathrm{BC}}$. Implementing the same approach as in **case 1,** the time of the collision becomes:

$$t_c = d_P \frac{\sin\phi_1 \sin(\phi_2 - \phi_3)}{v_1 \sin(\phi_2 - \phi_3) - v_2 \sin(\phi_1 - \phi_3) + v_3 \sin(\phi_1 - \phi_2)} \quad (1.23).$$

Left chiral line is constructed in the similar way as before, just for the lines $\overline{\mathrm{BC}}$ and $\overline{\mathrm{CA}}$ one gets (**Fig. S4(b)**):

$$s_{\overline{\mathrm{CA}}} = \frac{b_x - c_x + s_{\overline{\mathrm{BC}}}(c_x - b_x)}{(a_x - c_x)} \quad (1.24),$$

$$s_{\overline{\mathrm{BC}}} = \frac{\left(\frac{d_P}{t} - b_y + c_y\right)(a_x - c_x) + (b_x - c_x)(a_y - c_y)}{(c_y - b_y)(a_x - c_x) - (c_x - b_x)(a_y - c_y)} \quad (1.25),$$

$$\text{LDL:} \quad \begin{aligned} x &= \mathrm{C}_x - d_P \frac{\sin\phi_1 \sin\phi_2}{\sin(\phi_2 - \phi_1)} \\ y &= \mathrm{C}_y + d_P \frac{\cos\phi_1 \sin\phi_2}{\sin(\phi_2 - \phi_1)} \end{aligned} \quad (1.26),$$

and for the right chiral line, the same results as eqs. 1.19, 1.20 and 1.21 apply. Directions in which the chiral lines spread are the following:

LDL: **RDL**:

$$\alpha_{ll} = \mathrm{atan}\frac{y(t+dt)-y(t)}{x(t+dt)-x(t)} = \mathrm{atan}\frac{c_y}{c_x} \quad \alpha_{rl} = \mathrm{atan}\frac{y(t+dt)-y(t)}{x(t+dt)-x(t)} = \mathrm{atan}\frac{b_y}{b_x} \quad (1.22).$$

All possible chiral lines (except scenarios when one side of the domain spreads in the direction parallel to the long axis of the NT) are now expressed with the eqs. 1.17, 1.21 and 1.26.

Basic scenario considers WS_2 domain spreading with constant speed in all directions. In this case, left and right chiral lines are always $120°$ apart of each other. This can be partially modified considering the different speed of spreading of the domain sides. Let the speed of the spreading in x direction be vd_x and the speed of spreading in y direction be vd_y. If the elliptical speed dependency is chosen, then speed of the domain sides growth follows:

$$v_i = \frac{vd_x vd_y}{\sqrt{vd_y^2\cos^2\phi_i + vd_x^2\sin^2\phi_i}} = \frac{vd_y}{\sqrt{v_{ry}^2\cos^2\phi_i + \sin^2\phi_i}} = \frac{vd_x}{\sqrt{\cos^2\phi_i + v_{rx}^2\sin^2\phi_i}} \quad (1.23)$$

where $v_{ry} = vd_y/vd_x$ and $v_{rx} = vd_x/vd_y$.

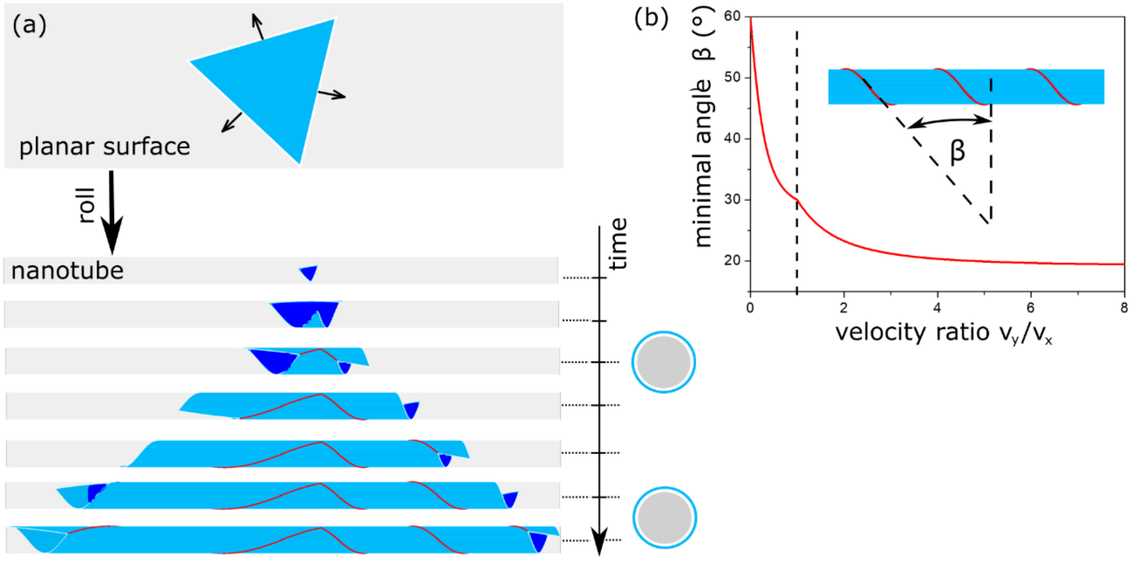

Fig. S5: Formation of a grain boundary line defect on a tubular template. Similar to Fig. 1 in the main text but with the second scenario. In (a) a theoretical model of lateral growth of a triangular domain on an unwrapped plain surface (top image) and on a tubular template is shown. Here, if two domain edges meet during growth, a grain boundary is formed instead of an edge. Cross-sections at different time intervals are shown as well. Like the first scenario in Fig. 1, the resulting line defect exhibits a chiral angle β. Importantly, this scenario does not permit formation of all chiral angles; there exists a certain minimal one. This is true even if the model accounts for anisotropic growth velocities (see model details above and Fig. S6). The dependence of minimal chiral angle β on the ratio of the two anisotropic growth velocities is shown in (b). Dashed vertical line marks ratio 1 (isotropic growth).

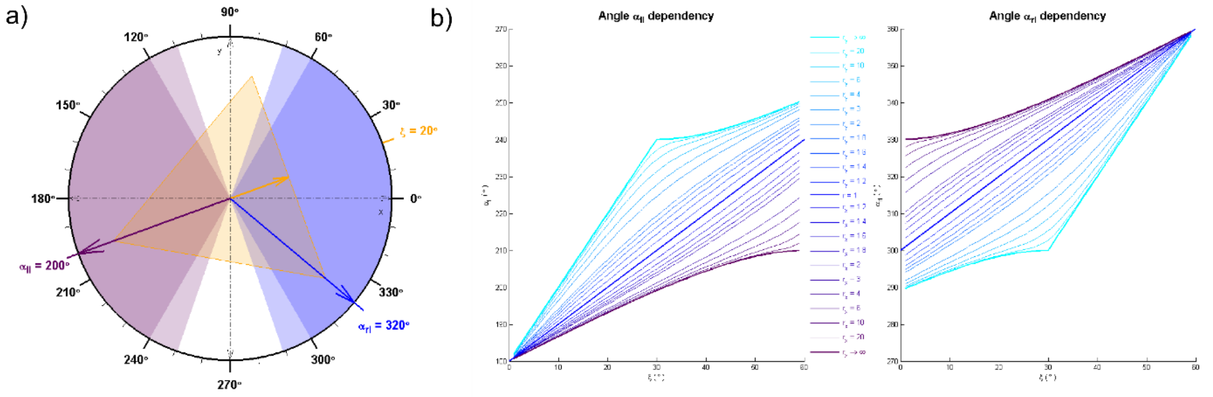

Fig. S6: Dependency of the chiral angle on the rotation of the domain (for chiral angle definition, see Fig. 1e in the main text). **a)** shows single WS_2 domain (orange triangle) spreading on a cylinder-like surface of the WS_2 nanotube (not shown). Due to a cylindrical symmetry, the spreading domain inevitably collides with itself, possibly creating a chiral line along this boundary. In coordinate system presented in a), an x-axis is parallel to the long axis of the nanotube and y-axis perpendicular to it. Rotation angle of the domain ξ is defined as shown: 0° corresponds to the case when one side of the equilateral-triangle-like domain is perpendicular to the x-axis. Due to symmetry of the presented model (the sides of the domain are indistinguishable from each other), the domain rotation angles from intervals (120°, 240°) and (240°, 360°) give the same results as ones from interval (0°, 120°). Moreover, symmetry of the problem allows as to focus only on interval of the domain rotation angles (0°, 60°), where results for all other domain rotation angles can be deduced. Figure a) shows example, where domain rotation angle is 20° and directions in which two chiral lines will be created (purple and blue arrow). Angles (directions) of these chiral lines are labelled as left chiral line α_{ll} and right chiral line α_{rl} (note that one chiral line will spread to the left and the other will spread to the right). If the domain spreads with constant speed in all directions, the angle between the α_{ll} and α_{rl} will always be 120°. Dark purple (respectively blue) painted area shows all possible α_{ll} (respectively α_{rl}) directions, in which a respective chiral line will spread (bearing in mind that directions of the two chiral lines need to be 120° apart). Figure a) together with b) also shows a case when domain is spreading with different speed in x-direction (direction parallel to the x-axis of the nanotube) and y direction (direction perpendicular to the x-axis of the nanotube). This is possible due to the different nanotube curvature in these directions. Within the model the investigated velocity dependency is elliptical with v_x and v_y component and corresponding ratios $r_x = v_x / v_y$ and $r_y = v_y / v_x$. Brighter purple (blue) painted area in figure a) shows additional possibilities for α_{ll} (α_{rl}) directions, in which a respective chiral line can spread for all possible speed ratios r_x and r_y. In this case, two chiral lines no longer need to be 120° apart and relation between them is more complicated (can be acquired from figure b). **b)** shows dependency of α_{ll} and α_{rl} chiral lines directions on rotation angle of the domain for different speed ratios. The empty (white) area in a) together with graphs in b) and c) indicate that not all chiral lines directions are achievable, despite the anisotropy in the velocity of the domain spreading.

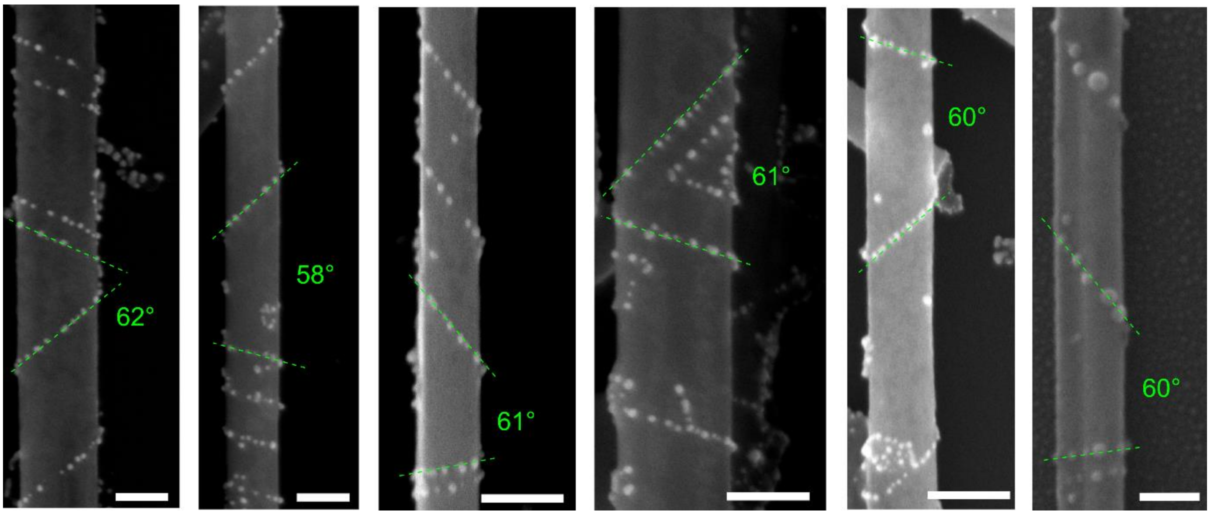

Fig. S7: Outer layer morphology visualized by gold nanoparticle attachment. The two chiral angles, as measured from the nanoparticle-decorated chiral lines, always exhibit ~60° in between. Such structure results from rolling of a triangular WS_2 sheet around a tubular template, supporting the TEM observation in Fig. 2 in the main article.

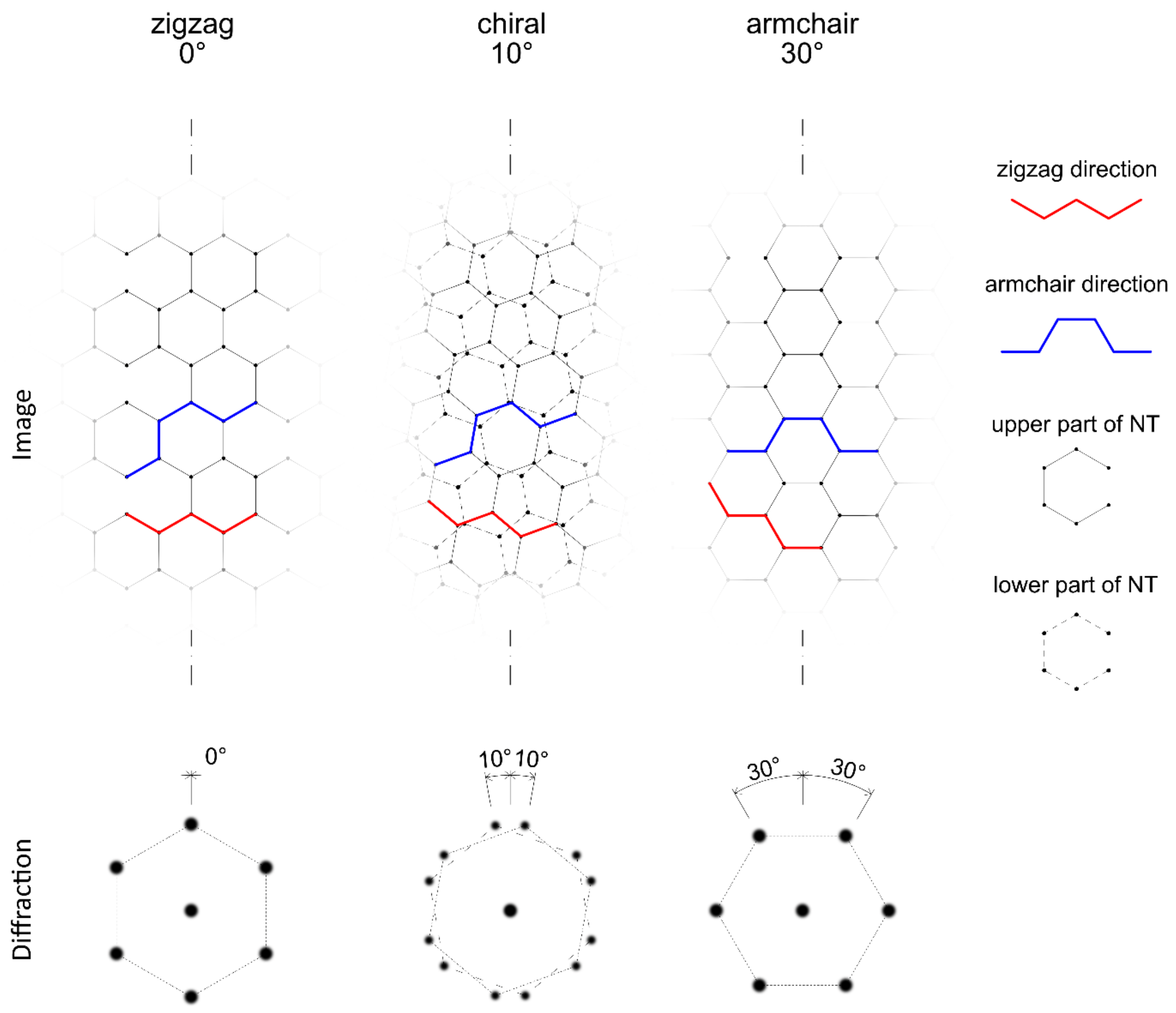

Fig. S8: Explanation of TEM analysis of nanotubes' chirality. For clarity, the images show hexagonal lattice and do not distinguish W and S atoms within the lattice. Nanotube axis is marked by a dash-and-dot line, zigzag edge is highlighted in red, armchair in blue. Nanotubes can be zigzag (left; zigzag edge is perpendicular to the nanotube axis), armchair (right) or generally chiral (middle). When viewed from top (like in TEM), the top and bottom layers lay just below each other for both zigzag and armchair nanotubes. This is not the case for chiral nanotubes, where these layers are twisted with respect to each other (by $2\times(90° - \beta)$, where β is the chiral angle defined in Fig. 1). This is clearly seen also in diffraction images (bottom line). The chiral angle of the nanotube can be determined from the twist angle between the hexagons in the diffraction image. If the outer layer is not complete (does not form a full monolayer, like in the case of a scroll), exposing a single step-edge, the comparison of diffraction image with a real space image of the nanoparticle-decorated step-edges allows to determine the edge termination. For specific chiral angle, only certain zigzag or armchair edge orientations are possible. For the case above (nanotube chiral angle 10°), a zigzag-terminated edge will exhibit −10° angle (derived from a nanoparticle chain), while armchair +20° (difference between both is fixed at 30°). For several examples, see table below.

Table S1: Determination of chirality of the outer layer and step-edge termination. The table shows inclination angles deduced from bright field images and chiral angles from the diffraction. The helical angle of nanoparticle chain deduced from a bright field image does not give enough information for chiral angle determination; the analysis results in two possible chiral angles in the range <−30°; 30°>, one for each termination (zigzag or armchair). Analysis of diffraction images provides several chiral angles, due to the fact that layers forming a nanotube can have different handedness. Indeed, this means that the chirality of the outer layer cannot be determined from, e.g., intensity of the diffraction spot. Here, combining bright field imaging and diffraction we look for the agreement between the chiral angle deduced by each method. This allows to determine the outer layer's chirality (green in 'diffraction image' column), but also its edge termination (green in 'bright field image' column). Note that the latter cannot be always determined (see nanotubes 1 and 3), but all the identified ones exhibit zigzag edge, which is agreement with other reports on edge termination of WS_2 (Ref. 56 in the main article). Also note that the angles deduced from the bright field images are always positive, as we cannot distinguish handedness from a single TEM image only. The exception here is nanotube no. 9, where subsequent analysis by SEM helped to determine handedness as well. Nevertheless, handedness is not necessary for step edge termination determination by this procedure.

NT no.	Chiral angle deduced from		
	bright field image		diffraction image
	zigzag	armchair	
1	**30**	0	0, 6, 11, 15, **30**
2	**30**	0	17, 25, 29, **30**
3	**16**	14	6, **17**
4	**8**	22	0, **6**, 17, 30
5	**17**	13	**16**, 30
6	**12**	18	0, 7, **14**, 21, 30
7	**25**	5	16, **23**, 30
8	**30**	0	30
9	**21**	-9	5, 17, **21**, 25, 30

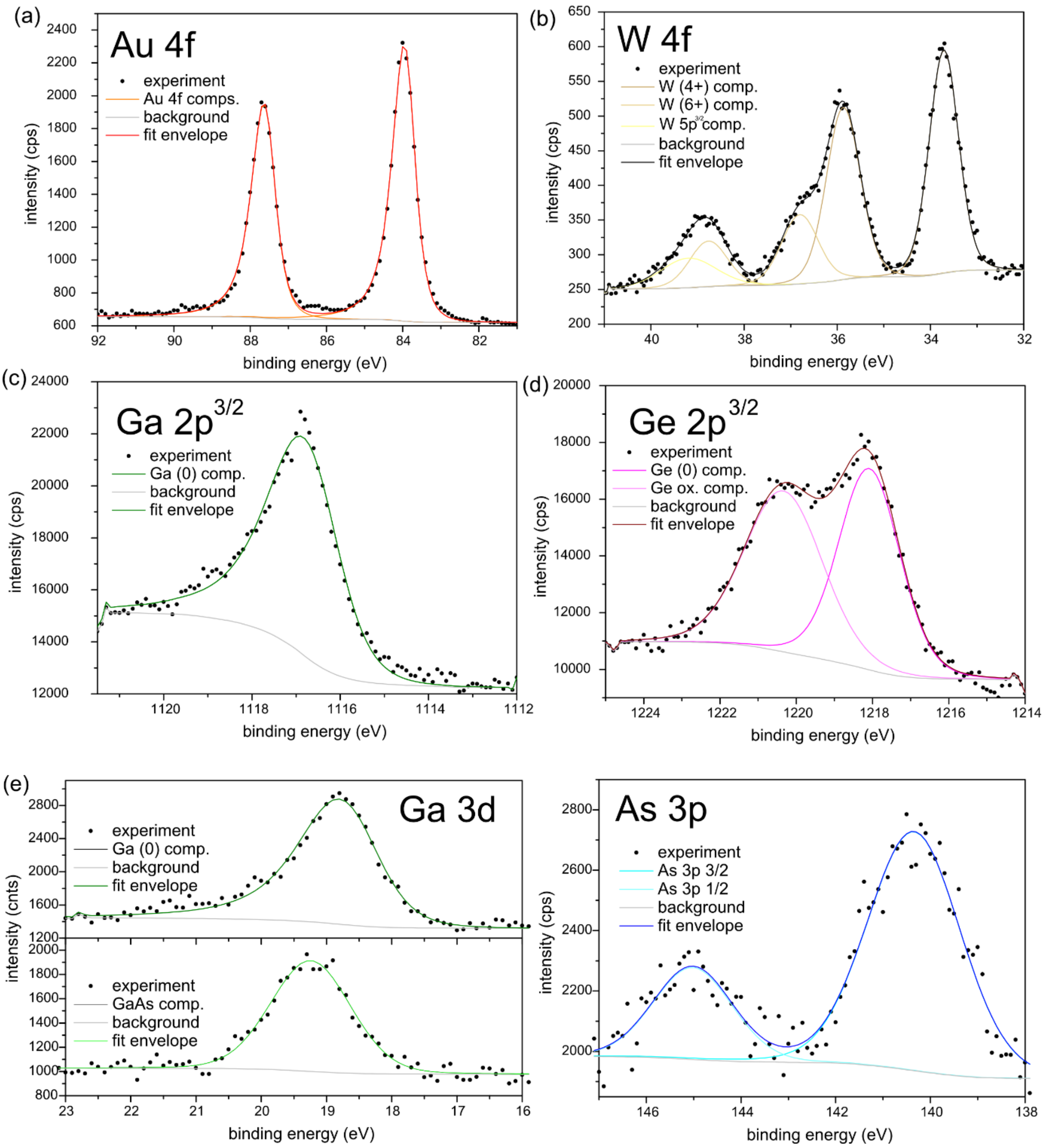

Fig. S9: Chemical state of the nanoparticles shown in Fig. 3 and XPS analysis of GaAs nanoparticles prepared by droplet epitaxy. Relevant XPS peak details acquired on samples shown in Fig. 3 are shown in (a)-(d). The experimental and peak fitting details are discussed in the text below. The chemical state of the nanoparticles can be inferred from the particular components of each peak: gold (a) and gallium (c) are in a metallic state, as they can be fitted by a single asymmetric component. The tungsten W 4f dublet (b) consists of two components. The more intensive one corresponds to W^{4+} in WS_2, while the other one to W^{6+} in WO_3. Note that this second component arises after the oxidation treatment and is absent for bare WS_2 nanotubes (ref. 57 in the main article). Hence, it can be assigned to the nanoparticles, which had emerged after the oxidation process. Lastly, Ge 2p peak (d) consists of two components. The higher-binding-energy one can be ascribed to native germanium oxide, which has resulted from the exposure of the nanoparticles to atmospheric conditions during transfer to Kratos XPS. Note that this is not the case for Ga, since this sample was measured without breaking

the vacuum conditions. In (e), an XPS analysis of Ga nanoparticles (c) exposed to As beam flux is shown, demonstrating the formation of GaAs via the Ga 3d peak line shape change and shift towards higher binding energy. The peak shift and the binding energy correspond to the tabulated values for GaAs.[S1,S2]

[S1] Ghosh, S.C.; Biesinger, M.C.; LaPierre, R.R.; Kruse, P. X-ray photoelectron spectroscopic study of the formation of catalytic gold nanoparticles on ultraviolet-ozone oxidized GaAs(100) substrates. *J. Appl. Phys*. **2007**, *101*, 114322. DOI: https://doi.org/10.1063/1.2743729
[S2] Budz, H.A.; Biesinger, M.C.; LaPierre, R.R. Passivation of GaAs by octadecanethiol self-assembled monolayers deposited from liquid and vapor phases. *J. Vac. Sci. Technol. B* **2009**, *27*(2), 637. DOI: https://doi.org/10.1116/1.3100266.

Details of the XPS measurements

The XPS analysis was performed either on a Kratos Analytical Axis Supra XPS or SPECS Phoibos 150. Monochromated Al Kα source was used in both cases. No charge neutralization was used; the stability of the measurement on Kratos Analytical tool was checked periodically by measuring C 1s peak (peak position was stable within ±0.1 eV). Detailed spectra were acquired in high magnification mode using pass energy of 20 eV (25 eV on SPECS), integrating at least 5 sweeps with 300-400 ms dwell time and 0.05-0.1 eV energy step. All the spectra were collected in normal emission geometry (emission angle parallel with surface normal).

The spectra were processed using Casa XPS. They were not shifted. Fitting details can be found in the table below (BE – binding energy, shift – with respect to the major component (with the lowest binding energy), dublet sep. – separation of the dublet peaks, FWHM – full-width-at-half-maximum, line shape – Casa line shape that was used for fitting).

	peak assignment	BE (eV)	shift (eV)	dublet sep. (eV)	FWHM(eV)	line shape
a)	Au 4f 7/2	83.9	-	3.7	0.65	LA(1.33,239)
b)	W 4f 4+ (WS_2)	33.7	-	1.1	0.8±0.05	GL(30)
	W 4f 6+ (WO_3)	36.8	3.1	1.0	0.95	GL(30)
	W 5p 3/2	39.4	5.7	-	1.4	GL(30)
c)	Ga 2p 3/2	1116.7	-	-	1.8	LA(1.33,2.43,69)
d)	Ge 2p 3/2	1218.1	-	-	1.8	GL(30)
	Ge 2p 3/2 (oxide)	1220.3	2.2	-	2.4	GL(30)
e)	Ga 3d (metal)	18.7	-	-	1.3	LA(1.33,2.43,69)
	Ga 3d (GaAs)	19.2	0.5	-	1.4	GL(30)
	As 3p 3/2	140.3	-	4.7	2.3	GL(30)
	As 3p 1/2	145.0	-	-	1.9	GL(30)

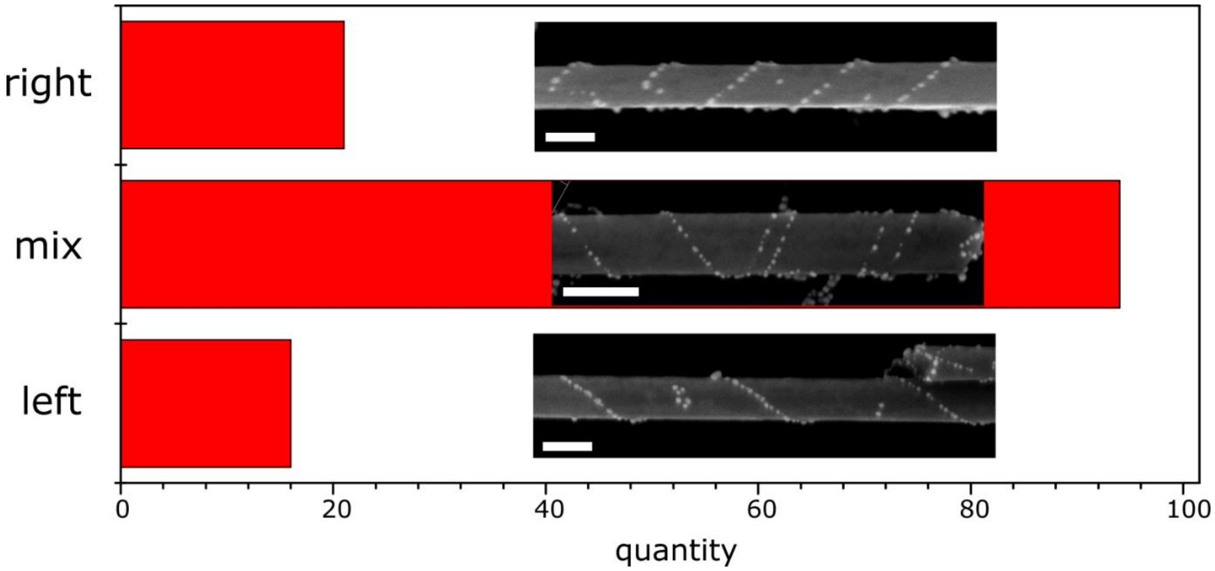

Fig. S10: Handedness of the nanoparticle helices. By viewing the nanoparticle-decorated NTs in an SEM, a handedness of the nanoparticle helices can be determined. Most of the nanotubes exhibit two helices of the opposite handedness, being an inherent property of the formation process (see Fig. 1 in the main text). Only a few nanotubes exhibit a single helix; the occurrence of left- and right-handed helices is almost the same. The resulting ensemble can be considered racemic. Scale bars are 100 nm.
